# Occurrence of the potent mutagens 2- nitrobenzanthrone and 3-nitrobenzanthrone in fine airborne particles

**DOI:** 10.1038/s41598-018-37186-2

**Published:** 2019-01-09

**Authors:** Aldenor G. Santos, Gisele O. da Rocha, Jailson B. de Andrade

**Affiliations:** 1 Instituto de Química, Universidade Federal da Bahia, Campus de Ondina, 40170-115 Salvador, BA Brazil; 2 Instituto Nacional de Ciência e Tecnologia em Energia e Ambiente - INCT, Universidade Federal da Bahia, 40170-115 Salvador, BA Brazil; 3 Centro Interdisciplinar em Energia e Ambiente - CIEnAm, Universidade Federal da Bahia, 40170-115 Salvador, BA Brazil; 4 SENAI-CIMATEC University Center, 41650-110 Salvador, Bahia Brazil

## Abstract

Polycyclic aromatic compounds (PACs) are known due to their mutagenic activity. Among them, 2-nitrobenzanthrone (2-NBA) and 3-nitrobenzanthrone (3-NBA) are considered as two of the most potent mutagens found in atmospheric particles. In the present study 2-NBA, 3-NBA and selected PAHs and Nitro-PAHs were determined in fine particle samples (PM 2.5) collected in a bus station and an outdoor site. The fuel used by buses was a diesel-biodiesel (96:4) blend and light-duty vehicles run with any ethanol-to-gasoline proportion. The concentrations of 2-NBA and 3-NBA were, on average, under 14.8 µg g^−1^ and 4.39 µg g^−1^, respectively. In order to access the main sources and formation routes of these compounds, we performed ternary correlations and multivariate statistical analyses. The main sources for the studied compounds in the bus station were diesel/biodiesel exhaust followed by floor resuspension. In the coastal site, vehicular emission, photochemical formation and wood combustion were the main sources for 2-NBA and 3-NBA as well as the other PACs. Incremental lifetime cancer risk (ILCR) were calculated for both places, which presented low values, showing low cancer risk incidence although the ILCR values for the bus station were around 2.5 times higher than the ILCR from the coastal site.

## Introduction

Cancer is one of the major causes of morbidity and mortality globally. In 2012 new cancer cases accounted for about 14 million new cases, with 8.2 million deaths occurred throughout the world. From that, circa 1.69 million deaths in 2012 resulted from lung cancer. However, only less than one third of them were derived from tobacco smoke^1,2^, what indicates there are other routes contributing to lung cancer incidence. Additionally, it is expected the number of new cancer diagnoses to be risen by about 70% over the next two decades, possibly reaching 21.7 million people, and the prediction of 13 million cancer deaths in 2030^2,3^.

Most cancer results from the interaction of genetics and the environment. However, hereditary or genetic factors themselves only respond for less than 10% of all types of cancers^4,5^. The remainder is attributed to environmental factors, and among them physical, chemical or biological toxicants, as well as individual susceptibility^4,6^ acting to explain the large cancer incidence worldwide. Human environmental and occupational exposure to atmospheric pollutants may be one of the major causes of lung cancer since the main pathway to atmospheric carcinogenic exposition is through inhalation^6–8^. It is well known that energy is the single most important cause of emissions of all main pollutants, and air pollution is an energy problem^9^. Carcinogenic and/or mutagenic compounds occurring in vapor phase and atmospheric aerosols, such as unsubstituted polycyclic aromatic hydrocarbons (PAHs) and their nitrated and oxygenated derivatives (nitro-PAHs and oxy-PAHs, respectively) are of major concern in regard to the potential risk of causing cancer.

Nitro-aromatic polycyclic hydrocarbons (nitro-PAHs) are ubiquitous airborne particle contaminants, mainly originated from incomplete combustion or pyrolysis of organic matter^10,11^ and/or photochemically-formed in atmosphere^12^. Nitro-PAHs are persistent compounds^13^ generally regarded as direct-acting carcinogenic and/or mutagenic agents to humans or animals^13–17^. Even though the nitro-PAH levels are typically one order of magnitude smaller than their unsubstituted congeners in temperate or remote regions, some of their members present high direct-acting mutagenic and/or carcinogenic potency in bacterial and mammalian cells^10,13^. Representatives may be cited, such as mono- and dinitropyrenes, nitrofluoranthenes and nitroketones^18,19^.

Although the understanding of the exact mechanisms of cancer incidence derived from atmospheric aerosols remains mostly uncertain^7,20^, it may be pointed out the nitroketone species 2-nitrobenzanthrone (2-NBA) and 3-nitrobenzanthrone (3-NBA) may be important contributors. They are ubiquously present in atmospheric particle samples as well as it has been reported evidences they contribute to the induction of tumors in animal models^18–23^. 3-NBA is a potent bacterial mutagen generally found in diesel and gasoline directly-exhausted particles. The 3-NBA carcinogenicity is comparable to 1,8-dinitropyrene, which is one of the most potent mutagens^19,21–30^. Indeed, 3-NBA is likely to form adducts to DNA molecule^19,24,25,28^ augmenting its genotoxic potential in living beings. In turn, the isomer 2-NBA is rather an ambient PM contaminant which is likely to be produced from the reaction of its precursor (benzanthrone, BA) with nitrogen oxides or other oxidants under typical atmospheric conditions^24–27,31–33^.

Despite the fact 2-NBA has been more abundantly found in airborne samples, screening assays studies suggest the genotoxic potency of 2-NBA is significantly lower than 3-NBA^34–36^. Both of them are lipophilic substances (K_ow_ = 3.99 for 2-NBA and K_ow_ = 3.90 for 3-NBA) yet they may be considered persistent in the environment^30^ and may be transferred from atmosphere to other environmental compartments by wet or dry deposition. Although their inherent relevance, there is limited information about the occurrence of 2-NBA and 3-NBA in aerosol particles.

2-NBA and 3-NBA have been unevenly and eventually identified in diesel exhaust and ambient air PM worldwide, although they have remained predominantly underdetermined. In part, the reason for finding little information about particle-bound 2-NBA and 3-NBA are their very low concentration levels in atmospheric aerosols (from low ng m^−3^ to low fg m^−3^), ranging from 0.5–3.5 f mol m^−3^ (or 0.14–0.96 pg m^−3^) (3-NBA) in Central Tokyo^22^ to 6.79 pg m^−3^ (3-NBA) in other parts of Japan^37^. Even fewer studies have considered 2-NBA. The reported concentration range of 2-NBA in ambient PM is around 49–831 f mol m^−3^ (or 13.5–229 pg m^−3^)^38^. Their low atmospheric concentrations together to the complex nature of particulate matter demand reliable and efficient sample preparation and analysis methods in order to be able to confidently quantify 2-NBA and 3-NBA in atmospheric samples. Nonetheless, the latest studies regarding 2-NBA and 3-NBA occurrence in atmospheric aerosols and rainwater and possible atmospheric chemistry implications are dated from late 1990s and early and mid 2000s only^22,26,27,30,32,37,39,40^. After that very little has been done in this subject. Consequently, the implications for atmospheric chemistry and their health-related endpoints are underestimated. More studies regarding the 2-NBA and 3-NBA in airborne particles are needed for better understand their role in these fields.

In the present study, we determined 2-NBA and 3-NBA in order to study the atmospheric occurrence of these species in ambient PM2.5 samples collected from a coastal tropical site in Northeastern Brazil as well as in samples collected in an underground level of a bus station, where buses exhausted mixtures of biodiesel to fossil diesel (B4) combustion during commuting. Together to that we also report some PAH levels in order to help trace atmospheric sources, which may be contributing to the found levels of 2-NBA and 3-NBA in our study. To date, this is the first time 2-NBA and 3-NBA levels are reported in the Southern Hemisphere. Cancer risk and mutagenic risk assessments from inhalation exposure were also calculated. Results are conveniently presented and critically discussed.

## Results and Discussion

### Analysis and identification of 2-NBA and 3-NBA

2-NBA and 3-NBA have been poorly determined in atmospheric aerosols. Their low atmospheric concentrations together to the complex nature of particulate matter demand reliable and efficient sample preparation and analysis methods in order to be able to confidently quantify 2-NBA and 3-NBA in atmospheric samples. In the same way, previous studies regarding 3-NBA and isomers were mostly determined via a derivatization step prior their analysis. Generally, the 2-NBA and 3-NBA derivatization is done through a reduction step which yields 2-aminobenzanthrone and 3-aminobenzanthrone (2-ABA and 3-ABA), respectively. They are further mostly analyzed by HPLC coupled with either fluorescence^22,41,42^, UV^22,39,41–43^, chemiluminescence^37,38,44^ and/or mass spectrometer^41^ detectors. If we consider possible losses or degradation as well as any artifact formations during 2-NBA and 3-NBA derivatization step associated to their very low atmospheric levels all together also may partially answer for the difficulty of finding them in appreciable levels in the atmospheric environment. Recently, *Santos et al*.^45^, reported, in a companion paper, a novel miniaturized method for the efficient determination of polycyclic aromatic compounds, and among them 3-NBA, by GC-MS with no derivatization or fractionation steps needed. Details about the sample preparation method is described in the Supplementary Information.

**Figure 1:**
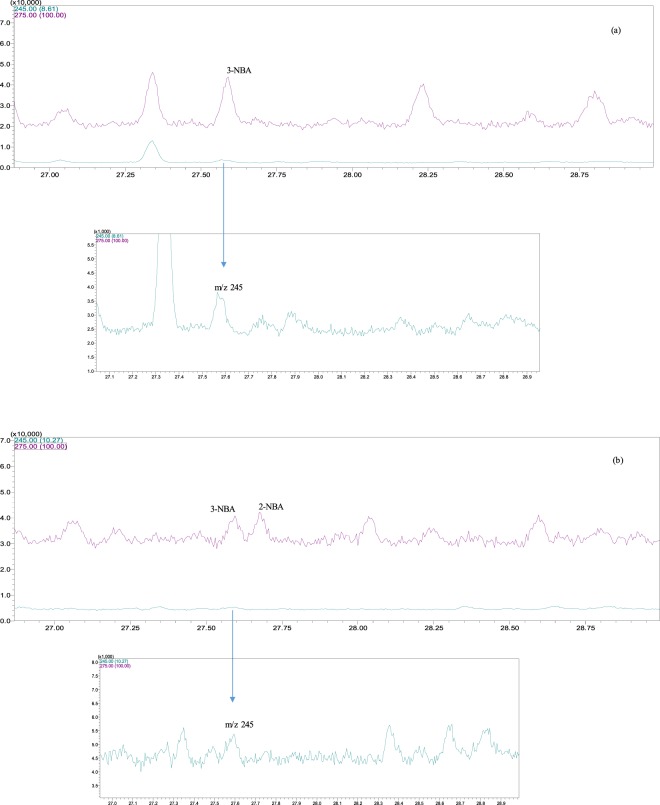
Chromatograms of 3-NBA and 2-NBA. (**a**) Bus station real PM sample, (**b**) ambient particulate sample from the coastal site. Limits of detection are 2.0 pg and 2.4 pg for 2-NBA and 3-NBA, respectively.

**Figure 2:**
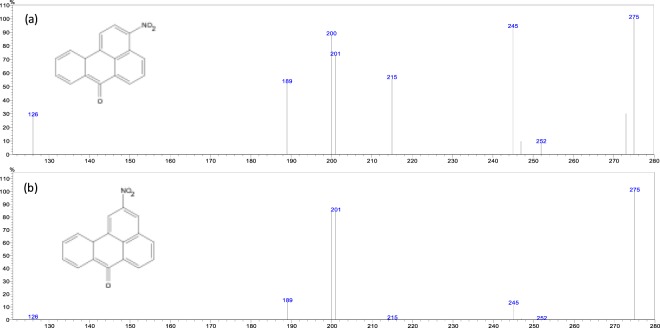
Electron impact mass spectra of (**a**) 3-nitrobenzanthrone and (**b**) 2-nitrobenzanthrone.

In the GC-MS system used in the present study 3-NBA is eluted just before 2-NBA, and both of them with retention times between 27.50 min and 27.75 min, as stated in Fig. 1. In this figure, we show two chromatograms of real samples, being Fig. 1a a chromatogram of a real PM2.5 sample from the bus station as well as Fig. 1b being a chromatogram of a real PM2.5 sample from the coastal site considered here. Accordingly, Fig. 2 shows the mass spectra of both 2-NBA and 3-NBA obtained in this study, which are in accordance with *Phousongphoung and Arey*^27^.

As stated in *Santos et al*.^45^, every analyte was monitored in SIM mode by three different *m/z* ions, with the addition of 2-NBA in the present study. Indeed, when our group published the results from *Santos et al*.^45^, 2-NBA was not listed as an analyte since we did not have its authentic analytical grade standard at that time. Nonetheless, in the current study we have added the 2-NBA to our mixed analytical standard solution and we could also quantify it in real samples without any modification of the GC-MS method. In order to distinguish 2-NBA from 3-NBA, we monitored three *m/z* ions, the ion base and two reference ions, in order to approach unequivocal identification. In this way, 2-NBA was identified (and differentiated from 3-NBA) by using *m/z* 275 (ion base) and *m/z* 201 and 245 (reference ions), as shown in detail in Fig. 1. In turn, 3-NBA was identified by *m/z* 275 (ion base) and *m/z* 215 and 245 (reference ions). Quantification was further done by considering the ion base signal only. Limits of detection (LOD) and limits of quantification (LOQ) were calculated from the calibration curve data. We considered *LOD = 3 s/a* and *LOQ* = *10 s/a*, where *“s”* is the standard deviation of the linear coefficient *“b”* and *“a”* is the angular coefficient (inclination) from calibration curve (in the format y = ax + b)^45^. LOD and LOQ concentrations values were converted to the minimum absolute mass either detected (LOD) or quantified (LOQ) by the MS in 1 µL standard solution injected in the GC-MS. LOD in terms of absolute mass, were 2.0 pg and 2.4 pg for 2-NBA and 3-NBA, respectively. Limit of quantification (LOQ) were 6.6 pg (2-NBA) and 8.1 pg (3-NBA). Finally, recovery levels were above 95% for both compounds. In this way, we consider our analytical methodology is adequate for studying 2-NBA and 3-NBA in the atmospheric environment.

### Occurrence of 2-NBA and 3-NBA associated to fine particles

**Figure 3:**
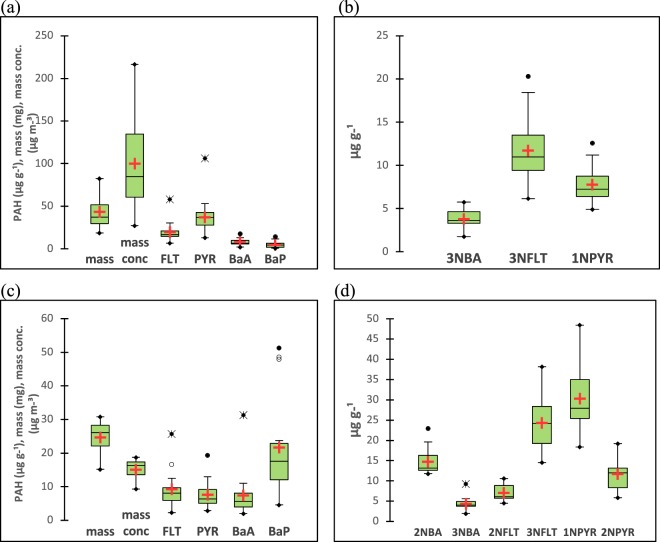
Box-and-Whisker plots for selected nitro-PAHs and PAHs in PM2.5, (**a**,**b**) diesel exhaust directly-emitted levels (bus station) and (**c**,**d**) ambient levels (coastal area). In this figure mean values are indicated by the red crosses, central horizontal bars are the medians, and the box lower and upper limits are the 1^st^ and 3^rd^ quartiles, respectively. Open circles and asterisks are outliers and closed circles are minimum/maximum values. The box plot width has no statistical meaning.

Both 2-NBA and 3-NBA were found in PM2.5 samples collected in the bus station and coastal site (Table 1 and Fig. 3). 3-NBA and 2-NBA concentrations (±one standard deviation) were calculated as pg m^−3^ (*picograms per cubic meter*) and as mixing ratios, in terms of µg g^−1^ (*micrograms per grams of particles*). We decided to do in this way since the former considers the total sampled volume air (or a normalization of the mass of 3-NBA or 2-NBA by total sampled volume air) while the latter consider the compounds masses normalized by the collected PM2.5 masses. We consider the measurement of 3-NBA and 2- NBA in terms of µg g^−1^ more useful for some of the discussions done here since it better represents the intrinsic or inherent characteristics of the PM considered in relation to 2-NBA and/or 3-NBA and makes sites with different levels of particle mass concentrations (due to different emission rates among sources) more directly and easily comparable. This is also advantageous to use this type of concentration unit when trying to address toxicological responses from those species possibly present in PM. Accordingly, we still present our results in terms of pg m^−3^ since it is needed when comparing our study with other reports found in the literature (Supplementary Information (SI) Table S1) and for risk assessment calculations (ILCR). In this study 3-NBA concentrations were 431 (±183) pg m^−3^ in the bus station and 59.0 (±16.6) pg m^−3^ in the coastal site. In turn, 2-NBA concentration was 200 (±18.8) pg m^−3^, although it was found in the coastal site samples only. Indeed, this is consistent with the fact 2-NBA is majorly formed photochemically and, therefore, it would not be found in the bus station samples (since this is a nearly indoor site mainly impacted by direct, freshly emitted vehicular particles). On the other hand, 3-NBA was found in both places, which is mainly derived from fuel burning/vehicular fleet present in these sites.

**Table 1:** PM2.5 mass concentrations (µg m^−3^), selected nitro-PAH and PAH concentrations (pg m^−3^) and mixing ratios (µg g^−1^).

*Compound*	*bus station (n = 19)*			*coastal site (n = 15)*		
	*pg m* ^*−3*^	*µg g* ^*−1*^	*Frequency of detection (%)*	*pg m* ^*−3*^	*µg g* ^*−1*^	*Frequency of detection (%)*
mass conc. (µg m^−3^)	91.5 ± 48.2	—	100	14.9 ± 2.88	—	100
***nitro-PAH***						
1-NPYR	1018 ± 527	7.81 ± 2.04	100	414 ± 108	30.3 ± 8.40	100
2-NPYR	385 ± 183^b^	2.82 ± 0.58	36	157 ± 44.1	11.7 ± 4.14	100
2-NFLT	378 ± 194^b^	2.47 ± 0.93	36	95.0 ± 20.4	7.06 ± 2.08	100
3-NFLT	1305 ± 395	11.8 ± 3.95	100	325 ± 49.1	24.3 ± 7.16	100
2-NBA	nd^a^	nd	0	200 ± 18.8	14.8 ± 3.36	100
3-NBA	431 ± 183	3.78 ± 1.13	100	59.0 ± 16.6	4.39 ± 1.64	100
***PAH***						
FLT	2025 ± 949	19.6 ± 11.0	100	122 ± 59.5	9.24 ± 5.77	100
PYR	3835 ± 2217	37.3 ± 19.7	100	102 ± 44.8	7.71 ± 4.27	100
BaP	522 ± 510	4.91 ± 3.80	100	0.32 ± 0.26	21.7 ± 15.3	100
BaA	853 ± 428	8.06 ± 3.72	100	75.4 ± 29.4	7.44 ± 7.12	100

Values are stated as mean ± standard deviation.

^a^Not detected.

^b^Since 2-NPYR and 2-NFLT is mainly produced in the atmosphere via photochemical reactions, they are uneven detected in the bus station samples. For the same reason, 2-NPYR and 2-NFLT are further disconsidered for the following calculations found in the present study.

The present study focuses on the determination of 2-NBA and 3-NBA in PM2.5 samples together to a better understanding of their atmospheric and human health implications. Other related and important compounds (such as 1-NPYR, 2-NPYR, 2-NFLT, 3-NFLT, FLT, PYR, BaA, and BaP) are also considered in order to support discussion and possible conclusions in regard to PAH and Nitro-PAH reactivities and source identification. Our findings show the 2-NBA levels is only about 3.4 times higher than the 3-NBA levels (Table 1). However, the few studies reported both 2-NBA and 3-NBA levels showed 2-NBA is ~35–70 higher than 3-NBA (Table S1)^22^. In our study while 2-NBA concentrations are similar to the literature, 3-NBA levels seem to be higher. This may be happening for some reasons. Considering 3-NBA is mostly emitted during vehicle fuel burning, this may be a consequence from the differences in the Brazilian fuel composition with other places in the world. Indeed, the Brazilian gasoline is 22–26% ethanol, and diesel fuel was actually a mix of 4% (v v^−1^) biodiesel into mineral diesel. Yet light-duty vehicles in Brazil has been set from the manufacturer to run with any ethanol-to-gasoline proportion. So, the 3-NBA emission is likely to differ from any other country in the world. How much is the 3-NBA emission rate from different fuel compositions should be better addressed in future studies.

Another point is that previous 2-NBA or 3-NBA studies were done mainly in temperate regions, with climate conditions completely different from our sampling places. In the tropics, the higher ambient temperature and more effective sun incidence could either favor 2-NBA photochemical production or its degradation through photolysis. All those points stated here could be plausible reasons in our study the ratio 2-NBA/3-NBA is lower than seen in literature. Since there are quite a few reported studies regarding both 2-NBA and 3-NBA these differences may also be due to limited understanding about their role in the atmosphere.

To our best knowledge, this study is the first to find 2-NBA and 3-NBA in a tropical area around a coast, and also adjacent to a large city (Salvador city and Metropolitan area) as well as to find 3-NBA emitted in the exhaust of fossil diesel-biodiesel mixes (B4) under real conditions. Yet, this is also the first study to investigate these species in fine particles, which brings more direct concern in regard to health-related endpoints, than other reports because they considered larger PM fractions only (such as PM10 and TSP). In this way, our results (Table S1) are not directly comparable with the previous ambient^22,32,38,39,42,43,46–48^, nor chamber studies^26,27^, especially concerned to particle size, different concentrations units reported, the lack of information regarding sampling data and the broad sort of analytical methods used.

Some selected PAHs and nitro-PAHs levels are stated in Table 1 and Fig. 3 in order to be able to better discuss possible nitro-PAHs photochemical routes and trace 2-NBA and 3-NBA main sources. 1-NPYR, 2-NPYR, 2-NFLT, 3-NFLT, and 3-NBA concentrations determined within the bus station (Table 1 and Fig. 3) represent the primary emission while the 1-NPYR, 2-NPYR, 2-NFLT, 3-NFLT, 2-NBA and 3-NBA concentrations determined in the coastal area represent both the primary and secondary contributions. They may also depend on the concentrations of PAH precursors in this site.

Our reasoning in this study in using two different sites, a bus station and a coastal site, was to investigate the emission profiles (which and how much) selected polyaromatics are emitted to atmosphere. In this study, we chose to collect PM2.5 samples in the underground floor of a bus station in order to better understand 3-NBA and related species emissions from diesel/biodiesel burning under real conditions (as opposed to sometimes unrealistic dynamometer studies). On the other hand, in the coastal area, which is affected by different sources including diesel/biodiesel burning, our goal was to evaluate the relative contribution among different sources to the found PAHs and nitro-PAHs levels in PM2.5. Possible differences in the PM2.5 emission rates from the bus station to the coastal site are minimized when using concentrations in terms of µg g^−1^, which consider the polyaromatic masses (in µg) normalized by the collected PM2.5 masses (in g). Thereby, the observed differences in the PAHs and nitro-PAHs levels between the sites (Table 1) are mainly derived from different sources relative contributions in each case.

The concentrations of FLT and PYR observed in the present study, in the bus station (19.6 ± 11.0 and 37.3 ± 19.7 μg g^−1^, respectively) are higher than the concentrations of 2-NFLT (2.47 ± 0.93 μg g^−1^) and 3-NFLT (11.8 ± 3.95 μg g^−1^) as well as 1-NPYR (7.81 ± 2.04 μg g^−1^) and 2-NPYR (2.82 ± 0.58 μg g^−1^). However, 2-NFLT and 2-NPYR concentration levels in the bus station are considered estimates only since they were found in 36% of the samples (which is considered a low detection frequency). Considering 2-NFLT and 2-NPYR are mainly produced photochemically and the samples from the bus station were collected in the underground floor with absence of light, they were unevenly found in this site. In this way, 2-NFLT and 2-NPYR levels from the bus station is no further considered. Hence, the nitro-PAHs (and also PAHs) determined inside the bus station are not associated to the atmospheric reactions. Indeed, our results from the bus station show both PAHs and nitro-PAHs studied here are directly emitted by fossil diesel/biodiesel burning. On the other hand, the results determined in the coastal area showed an opposite trend, the nitro-PAH levels, [2-NFLT (7.06 ± 2.08 µg g^−1^) and 2-NPYR (11.7 ± 4.14 µg g^−1^)], are higher than the PAH precursors [FLT (9.24 ± 5.77 µg g^−1^) and PYR (7.71 ± 4.27 µg g^−1^)]. These results are in good agreement with PAHs reactivity since PYR is less reactive than FLT in nitration reactions^15,16,49–53^. Also, as can be seen by Fig. S1, air masses arriving the sampling site were subjected to long-range transport (oceanic origin). In fact, during the long-range transport probably PAH-rich air masses were gradually being transformed to their respective nitro-PAH congeners. Indeed, 2-NFLT is known to be formed via reaction between FLT and NO_3_ or OH radicals while 2-NPYR is the product of the reaction between PYR and OH radicals only^17,22,37,49–53^. This route seems to be important for tropical and warm areas, where OH radical formation is likely to be enhanced due to higher sunlight incidence. This implies in the coastal area there are important enrichment of 2-NFLT and 2-NPYR, both photochemically produced, in relation to directly-emitted FLT and PYR by automobiles, as observed when compared with their concentrations found in the bus station.

On the other hand, 2-NBA formation is mainly done via heterogeneous reaction between benzanthrone (not measured here) and NO_3_ or OH radicals on preexisting particles. According to *Abbas et al*.^54^, there are three different processes which may lead to nitro-PAHs formation from the parent PAHs. They can be formed by *(a)* electrophilic nitration *within the combustion process* (*e*.*g*. in the exhaust of diesel or gasoline vehicle engines, wood burning and cooking), *(b)* by gas-phase reactions with atmospheric oxidants (as a secondary and homogeneous process), and *(c)* by heterogeneous oxidation of particle-bound PAHs (also a secondary process). For instance, 3-NBA is mainly formed by the mechanism described by *(a)*. Only parent PAHs with less than 4 benzene rings and high vapor pressure would be substantially in gas phase in order to react with oxidants in typical atmospheric conditions to form their respective nitro-PAHs, as in *(b)*. Considering benzanthrone vapor pressure is low and boiling point is high (2.21 × 10^−7^ mm Hg and 403 °C, respectively) and log K_oa_ is high (10.378) (Table S2), this species would be principally present in PM rather than gas phase under typical atmospheric conditions. In this way, we argue 2-NBA main formation route would be via heterogeneous reactions. However, the 2-NBA formation through heterogeneous reaction still needs to be addressed in future studies.

In terms of source tracing, it is well accepted 2-NBA, 2-NFLT, and 2-NPYR are predominantly generated via photochemical reactions in the atmosphere while 3-NBA, 3-NFLT, and 1-NPYR is mainly emitted by diesel combustion/vehicles^17,22,37,53,54^. Here again, it implies photochemical reactions are important to explain the nitro-PAHs and PAHs atmospheric levels in the coastal area.

### Multivariate Analysis

**Figure 4:**
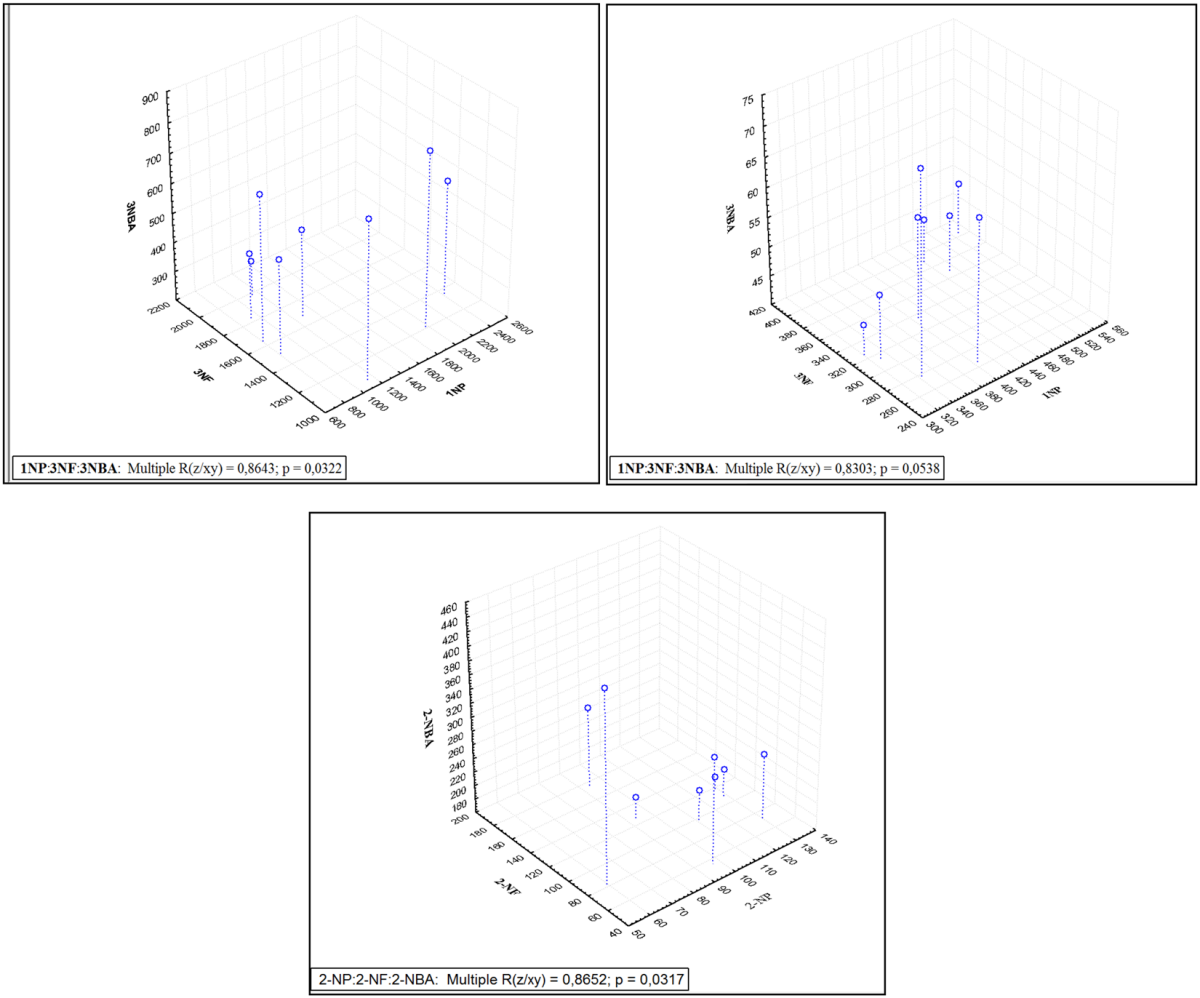
Ternary correlations among selected substances, top left: bus station, top right and bottom: coastal area.

Figure 4 shows ternary correlations, which are useful for improving source identification. For 1-NPYR, 3-NFLT, and 3-NBA we found high correlation (r = 0.8643, *p* = *0*.*0322*) for the bus station and (r = 0.8303, *p* = *0*.*0538*) for the coastal area. Since they are mainly directly emitted by diesel combustion, this high correlation implies this source is relevant for them in both sites. In turn, for 2-NPYR, 2-NFLT, and 2-NBA we also see high correlation (r = 0.8652, *p* = *0*.*0317*) for coastal area, which demonstrates photochemistry actually is an important source for this ambient site. Although in the second case the *p-value* is little higher than 0.05, indicating a statistical confidence slightly lower than 95%, we should keep in mind the *r-values* are high enough to be statistically valid when considering ternary correlations.

**Figure 5:**
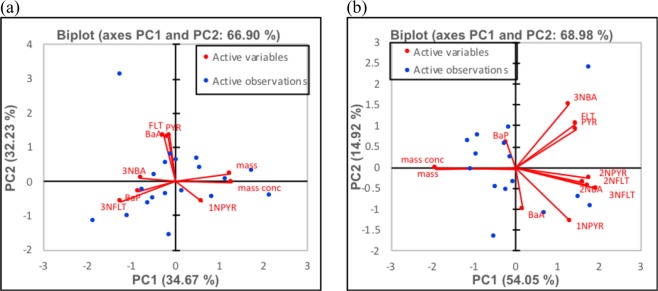
Principal component analysis among active variables (or species determined in the samples, red circles) and active observations (or sampling days, blue circles) at 95% confidence level, for (**a**) bus station and (**b**) coastal site.

Principal component analysis (PCA) were run for both sites (Fig. 5 and SI Table S3). For the bus station PCA explains 66.9% of the whole dataset variance. PC1 accounted for 34.7% of the variance, and it had high positive loadings for particle mass and mass concentration as well as high negative loading for 3-NFLT, and moderate negative loadings for 3-NBA, BaP, and 1-NPYR. In turn, PC2 explained 32.2% of the variance, with high positive loadings for FLT, PYR and BaA. PC1 seems to indicate diesel-biodiesel exhaust direct-emitted as source while PC2, which is represented by less reactive species, then they are able to be constituents of particles direct-emitted by buses and deposited on the floor and be suspended again, which may indicate particle aging and/or particle size growing processes happening in this site.

For the coastal site, it was necessary to consider three principal components to explain 82.6% of the total variance. PC1 explains 54.1%, with high negative loadings for particle mass and mass concentration as well as high positive loadings for most of the nitro-PAHs (2-NBA, 2-NFLT, 3-NFLT, 1-NPYR, and 2-NPYR), except for 3-NBA represented in the PC2 (which accounted for 14.9% of the variance), together to FLT and PYR. PC3 accounted for 13.6% of the variance and presents high positive loadings for BaP and BaA. For this site, PC1 represents the photochemical origin (as important secondary source tracers, such as 2-NBA, 2-NPYR and 2-NFLT are better explained in this PC). Although in PC1 3-NFLT and 1-NPYR, which are direct-emitted species, are also presented, it seems through particle aging, some isomers may interconvert into the other (2-NFLT to 3-NFLT and 1-NPYR to 2-NPYR or vice-versa) forming complex interactions. PC2 may indicate direct emission of fuel combustion as probable source since 3-NBA, PYR, and FLT are presented by this PC. Finally, PC3, which has high scores for BaA and BaP, is likely to represent wood combustion as sources.

**Figure 6:**
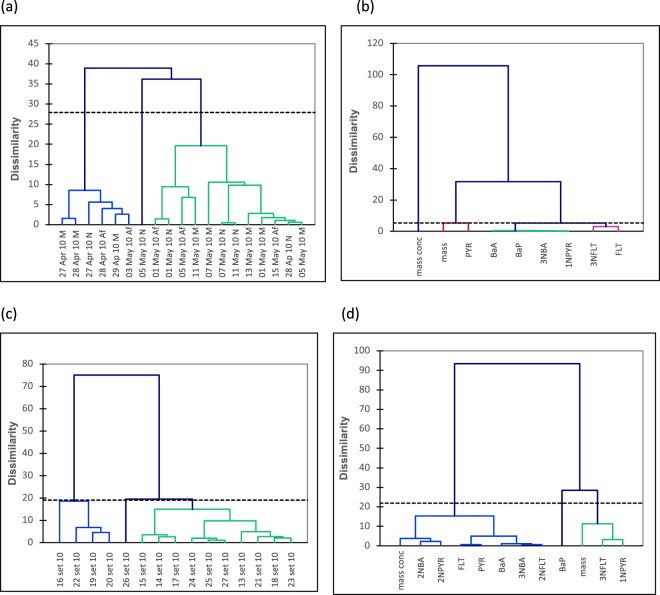
Agglomerative hierarchical clustering plots for (**a**,**b**) bus station and (**c**,**d**) coastal site datasets.

Agglomerative hierarchical clustering (AHC) plots (Fig. 6a,b) for the bus station does not show statistically significant dissimilarities for particles collected in the morning, afternoon or night periods nor among species (PAHs and nitro-PAHs). This is due to the fact the bus station does have few different sources contributing to the levels of PAHs and nitro-PAHs in fine particles there, as suggested by the PCA study. On the other hand, for the coastal site (Fig. 6c,d), in the AHC plots there are 3 clusters discriminated. The first cluster is formed by mass concentration, 2-NBA, 2-NPYR, FLT, PYR, BaA, 3-NBA, and 2-NFLT. In turn, the second cluster is only formed by BaP, and the last cluster includes particle mass, 3-NFLT, and 1-NPYR. The first cluster represents the parent PAHs and their nitro derivatives, while the second one is tentatively attributed to wood combustion. Finally, the last cluster is representative of primary emissions.

### Cancer risk from inhalation exposure

Estimates of incremental lifetime cancer risk (ILCR) according to four different age groups, namely infants (<1 year), children (1–11 years), adolescents (11–16 years), and adults (>21 years) in the population are summarized in Table 2. BaPeq considering the carcinogenicity contribution are 1.21 and 0.45 ng m^−3^ as well as BaPeq due to mutagenicity 0.90 and 0.38 ng m^−3^ for the bus station and coastal site, respectively. Despite the fact there are several studies showing 2-NBA and 3-NBA high levels of carcinogenicity and/or mutagenicity^11,13,18,21,25–27,29,30,36,55,56^ are similar to those ones of dinitropyrenes (which, in turn, are considered the most potent carcinogens and mutagens), there are no TEFs and MEFs for the nitrobenzanthrone isomers. At this point, knowing the 2-NBA and 3-NBA carcinogenicity and mutagenicity potencies and not considering them in the ILCR calculations would be a not acceptable underestimation for such relevant target species for human health. This would be interesting to account the nitrobenzanthrones in the ILCR since representing the air toxicity by considering only the 16 priority PAHs is not enough anymore in face to the recent advances in this area and the scientific community recent discussions^56,57^. Even though knowing this is not ideal, we have decided to tentatively use the 1,3-dinitropyrene (1,3-DNPYR) TEF and MEF values as surrogates for 2-NBA as well as 1,8-dinitropyrene (1,8-DNPYR) TEF and MEF as surrogates for 3-NBA (SI Table S4). In our point of view, this is plausible because the 1,3-DNPYR and 1,8-DNPYR carcinogenicity and mutagenicity in assays using mammalian cells, bacteria and rodents are close to the ones from 2-NBA and 3-NBA in the same screening assays^11,19,21,22,25–30^. After that we were able to calculate more realistic BaPeq values (Table 2). In this way, even though we are aware this may be just a proxy to their real values, we propose to adopt 1,8-DNPYR and 1,3-DNPYR TEF and MEF values as 2-NBA and 3-NBA TEF and MEF, respectively. This could be a temporary alternative while studies about evaluation of the real TEF and MEF for the nitrobenzanthrone isomers are not directly measured.

**Table 2:** Daily inhalation levels (E_I_) and incremental lifetime cancer risk (ILCR) in relation to carcinogenicity and mutagenicity for selected groups in the population.

Target population	BaP_eq_ (ng m^−3^)^a^		E_I_ (ng person^−1^ day^−1^)		ILCR	
	bus station	coastal site	bus station	coastal site	bus station	coastal site
***considering carcinogenicity only***						
Adults (>21 years)			19.9	7.34	5.57 × 10^−7^	2.06 × 10^−7^
Adolescents (11–16 years)			26.5	9.81	1.26 × 10^−7^	4.65 × 10^−8^
Children (1–11 years)	1.21	0.45	16.1	5.96	3.00 × 10^−7^	1.11 × 10^−7^
Infants (<1 year)			8.24	3.05	5.43 × 10^−8^	2.01 × 10^−8^
***considering mutagenicity only***						
Adults (>21 years)			14.8	6.30	4.16 × 10^−7^	1.77 × 10^−7^
Adolescents (11–16 years)			19.8	8.41	9.38 × 10^−8^	3.99 × 10^−8^
Children (1–11 years)	0.90	0.38	12.0	5.11	2.24 × 10^−7^	9.51 × 10^−8^
Infants (<1 year)			6.15	2.61	4.05 × 10^−8^	1.72 × 10^−8^
***carcinogenicity*** + ***mutagenicity***						
Adults (>21 years)			34.7	13.6	9.73 × 10^−7^	3.83 × 10^−7^
Adolescents (11–16 years)			46.3	18.2	2.17 × 10^−7^	8.64 × 10^−8^
Children (1–11 years)	2.11	0.83	28.1	11.1	5.24 × 10^−7^	2.06 × 10^−7^
Infants (<1 year)			14.4	5.66	9.48 × 10^−8^	3.73 × 10^−8^

^a^Results are expressed as average ± one standard deviation.

Bus station total daily inhalation exposure (E_I_) (considering both carcinogenicity and mutagenic contributions), in ng person^−1^ day^−1^ according to different age groups, were 14.4, 28.1, 46.3, and 34.7 for infants, children, adolescents and adults, respectively. Total ILCR was 9.48 × 10^−8^ (infants), 5.24 × 10^−7^ (children), 2.17 × 10^−7^ (adolescents), and 9.73 × 10^−7^ (adults). These ILCR estimates mean there is a risk of 9.48 infants in a hundred million and ranges up to 9.73 adults in ten million commuting this bus station to develop cancer during their lifetime.

In the coastal site total E_I_ were 5.66, 11.1, 18.2, and 13.6 ng person^−1^ day^−1^ for infants, children, adolescents, and adults respectively. Total ILCR were 3.73 × 10^−8^ (infants), 2.06 × 10^−7^ (children), 8.64 × 10^−8^ (adolescents), and 3.83 × 10^−7^ (adults). Coastal site ILCR is about 2.5 times shorter than the bus station ILCR. In the same way, ILCR estimates for this site means there is a chance of 3.73 infants in a hundred million and ranges up to 3.83 adults in a ten million that may get cancer during their 70 years of lifetime.

If we compare these ILCR to other recent studies^15,16,23,58–60^ our estimates could be considered one or two orders of magnitude lower, which primarily may not raise much concern. This is needed further studies in order to obtain more comprehensive ILCR estimates. On the other hand, it should be kept in mind these estimates only address ILCR from about ten polycyclic compounds in fine particles considered in this study. This is well accepted there are thousands of other compounds constituting fine particulate matter and we actually have investigated the large majority of them and much less is known about their toxicity in regard to carcinogenicity and mutagenicity.

## Concluding Remarks

Selected fine particulate PAHs and nitro-PAHs were characterized under typical conditions from a bus station and a coastal site. Among nitro-PAHs, 2-NBA and 3-NBA, which are potent carcinogens and mutagens were determined for the first time in the Southern Hemisphere. The main sources for the studied compounds in the bus station were mineral diesel/biodiesel exhaust followed by floor resuspension (which contributed to the particle growing and ageing). In the coastal site, vehicular emission, photochemical formation and wood combustion were the main sources for 2-NBA and 3-NBA as well as the other polycyclic aromatic compounds. Incremental lifetime cancer risk (ILCR) were calculated for both places, which presented low values, showing low cancer risk incidence although the ILCR values for the bus station were around 2.5 times higher than the ILCR from the coastal site.

## Methods

### Reagents and Standards

In this work, we used as authentic nitro-PAH standards a NIST SRM 2265 (polycyclic aromatic hydrocarbons nitrated in methylene chloride II), which contained 2-nitrofluoranthene (2-NFLT, CAS# 13177-29-2), 3-nitrofluoranthene (3-NFL, CAS# 892-21-7), 1-nitropyrene (1-NPYR, CAS# 5522-43-0), 2-nitropyrene (2-NPYR, CAS# 789-07-1), and 3-nitrobenzanthrone (3-NBA, CAS# 17117-34-9), among others. Their certified concentrations were 5.46 ± 0.15 µg mL^−1^ (2-NFLT), 6.14 ± 0.13 µg mL^−1^ (3-NFLT), 6.91 ± 0.27 µg mL^−1^ (1-NPYR), 6.91 ± 0.27 µg mL^−1^ (2-NPYR), and 4.39 ± 0.11 µg mL^−1^ (3-NBA). Since SRM 2265 does not include 2-nitrobenzanthrone (2-NBA, CAS# 111326-48-8), this compound was purchased from Sigma-Aldrich (USA) (>99% purity) and added to that. Authentic standards for fluoranthene (FLT, CAS# 206-44-0), pyrene (PYR, CAS# 129-00-0), benzo[a]pyrene (BaP, CAS# 50-32-8), and benzo[a]anthracene (BaA, CAS# 56-55-3), among others, are included in the EPA 610 PAH mix, at 2000 µg mL^−1^ each, in methanol: methylene chloride (1:1) (Supelco, USA). In this study, stock and analytical solutions were prepared by successive dilutions in acetonitrile (chromatographic and spectroscopic grade, J.T. Baker, USA).

### Instrumentation and analysis

In this study, we used the chromatographic conditions stated in *Santos et al*.^45^ Briefly describing, we utilized a high-resolution gas chromatograph-high-resolution mass spectrometer detector (HRGC-HRMS) from Shimadzu (GCMS-QP2010Plus, Shimadzu, Japan) with a Rtx-5MS gas capillary column (30 m × 0.250 mm × 0.25 µm, Restek Bellofonte, USA). Oven temperature programing initiated at 70 °C (2 min), then rising from 70–200 °C (30 °C min^−1^, 5 min), and 200–330 °C (5 °C min^−1^, 0.67 min). Injector temperature was set at 310 °C and transfer line was 280 °C. Analysis was done in GC-MS-SIM, at electron impact mode (EI) (70 eV). Sample preparation was done using a filter piece of 4.15 cm^2^ diameter added to a miniaturized micro-extraction device using 500 µL solvent extraction^45,61^. Sample preparation details are found in Supplementary Information.

### Sample collection

PM2.5 samples were collected in two different sites: *(i)* in the underground floor of a bus station (12°58′S, 38°30′W, 52 m altitude), and *(ii)* in a coastal area (12°58′S, 38°30′W, 52 m altitude) in Northeastern Brazil^62–64^. The PM samples collected in the bus station were mostly subjected to the exhausts from buses, which remained on (in idle point) while waiting for passengers. No substantial additional sources have contributed to the found levels of polycyclics since the underground level of this bus station is a nearly indoor environment. Yet, no air dispersion system was present. This bus station has been previously studied by our research group^45,61,62,64^ and our findings show PM sample sources mainly are biodiesel/diesel burning released by buses and dust resuspension. During the sampling period, buses were using the B4 mix as fuel (4% v v^−1^ biodiesel to fossil diesel, as established by law at that time). In turn, PM2.5 samples were collected around the Todos os Santos Bay, in the Brazilian Navy Base, located in Salvador Metropolitan Area, State of Bahia, Northeastern Brazil. This site is close to industries and the Aratu Harbor and it is also influenced by vehicular fleet from Salvador City and surroundings^62,64^. During sample collection, ambient temperature ranged from 22.5 to 24.9 °C, relative humidity was 72–84%, solar radiation was 145–340 W m^−2^, and wind speed ranged 3.9–8.0 m s^−1^ (Mkoma *et al*.^62^). Backward air mass trajectories were calculated starting 48 h before arrival time (00:00 UTC) and 500 m a.g.l. During the sampling time air mass trajectories were of typically oceanic origin passing through urban and industrial areas around the coast (Fig. S1).

PM_2.5_ samples were collected using a high-volume (Hi-Vol) sampler (Energetica, Brazil) with an inlet for classifying particles smaller than 2.5 μm aerodynamic diameter (Thermo Andersen, USA). Samples were collected on quartz microfiber filters (22.8 cm × 17.7 cm, Whatmann, USA) over 4–12 h periods (7 AM to 2 PM, 2 PM to 7 PM, and 7 PM to 7 AM, completing a 24 h period per day, at the bus station) or during 24 h at the coast, at 1.13 m^3^ min^−1^. The sampling campaigns lasted 15 consecutive days in each place. After collection, filters were folded in half face-to-face, placed in an aluminum foil envelope then in a zip lock type plastic bag, and finally placed in sealed plastic containers for avoiding any contamination. Following, samples were transported cool to the laboratory and stored in a freezer (−4 °C) until analysis. Field blanks also were considered in this study. Field blank filters were placed in the same containers where sample filters were transported from laboratory to the sampling site and back to laboratory in order to make any minor contamination, if there was any, traceable. Our procedure included analysis of both sample and field blank filters in exactly same way for having any detectable analyte signal in field blank discounted from sample filter results. In this work, we did not detect any analyte in the field blanks.

### Backward air mass trajectories and statistical analysis

Backward air mass trajectory frequencies were calculated during the coastal site sampling time by using the NOAA HYSPLIT database^65,66^. Trajectory frequencies (as number of endpoints per squared grid per number of trajectories) were calculated with frequency grid resolution 1.0° × 1.0°, starting 96 h before arrival time (00:00 UTC) and altitudes ranging from 0 to 99999 m a.g.l. During the sampling period, air mass trajectories were of typical oceanic contribution, passing on Atlantic Ocean through urbanized city centers and industrialized areas around the coast before arriving to our coastal sampling collection site (SI Fig. S1).

Multivariate statistical analyses, such as Pearson correlation, ternary correlation, Principal Component Analysis (PCA) and Agglomerative Hierarchical Clustering (AHC) were calculated for both dataset by using XLSTAT BASE software package version 19.5 for Microsoft Excel from Addinsoft Ltd (Paris, France). Ternary correlations were done by STATISTICA version 12.0 (Statsoft, USA).

### Incremental lifetime cancer risk assessment

Carcinogenic and mutagenic risk assessments^15,60–63,67–69^ induced by inhalation of PM2.5-bound enriched with selected nitro-PAHs (1-NPYR, 2-NPYR, 2-NFLT, 3-NFLT, 2-NBA, and 3-NBA) and PAHs (PYR, FLT, BaP, and BaA) were estimated in the bus station and coastal site samples according to calculations done by *Wang et al*.^60^, *Nascimento et al*.^61^, *and Schneider et al*.^67^ PAH and PAH derivatives risk assessment is done in terms of BaP toxicity, which is well established^67–73^. The daily inhalation levels (E_I_) were calculated as:

1 $${E}_{I}=Ba{P}_{eq}\times IR=(\sum {C}_{i}\times TE{F}_{i})\times IR$$

where *E*_*I*_ (ng person^−1^ day^−1^) is the daily inhalation exposure, *IR* (m³ d^−1^) is the inhalation rate (m³ d^−1^), *BaP*_*eq*_ is the equivalent of benzo[a]pyrene *(BaP*_*eq*_ = *Σ C*_*i*_ × *TEF*_*i*_) (in ng m^−3^), *C*_*i*_ is the PM2.5 concentration level for a target compound *i*, and *TEF*_*i*_ is the toxic equivalent factor of the compound *i*. TEF values were considered those from *Tomaz et al*.^15^, *Nisbet and LaGoy*^69^, *OEHHA*^72^, *Durant et al*.^73^, and references therein. E_I_ in terms of mutagenicity was calculated using equation (1), just replacing the TEF data by the mutagenic potency factors (MEFs) data, published by *Durant et al*.^73^. Individual TEFs and MEFs values and other data used in this study are described in SI, Table S4.

The incremental lifetime cancer risk (ILCR) was used to assess the inhalation risk for the population in the Greater Salvador, where the bus station and the coastal site are located. ILCR is calculated as:

2 $$ILCR=({E}_{I}\times SF\times {E}_{D}\times cf\times EF)/(AT\times BW)$$

where *SF* is the cancer slope factor of BaP, which was 3.14 (mg kg^−1^ d^−1^)^−1^ for inhalation exposure^60^, *EF* (day year^−1^) represents the exposure frequency (365 days year^−1^), *E*_*D*_ (year) represents exposure duration to air particles (year), *cf* is a conversion factor (1 × 10^−6^), *AT* (days) means the lifespan of carcinogens in 70 years (70 × 365 = 25,550 days)^70,72^, and *BW* (kg) is the body weight of a subject in a target population^71^.

The risk assessment was performed considering four different target groups in the population: adults (>21 years), adolescents (11–16 years), children (1–11 years), and infants (<1 year). The IR for adults, adolescents, children, and infants were 16.4, 21.9, 13.3, 6.8 m^3^ day^−1^, respectively. The BW was considered 80 kg for adults, 56.8 kg for adolescents, 26.5 kg for children and 6.8 kg for infants^70^.

## Supplementary information

Occurrence of the potent mutagens 2- nitrobenzanthrone and 3-nitrobenzanthrone in fine airborne particles
