# Supplementary material for: Occurrence of the potent mutagens 2- nitrobenzanthrone and 3-nitrobenzanthrone in fine airborne particles
Source: Sci Rep. 2019 Jan 9;9:1. doi: 10.1038/s41598-018-37186-2 (PMC6327027; doi:10.1038/s41598-018-37186-2)
Supplement: Supplementary file 1 — Occurrence of the potent mutagens 2- nitrobenzanthrone and 3-nitrobenzanthrone in fine airborne particles [file 41598_2018_37186_MOESM1_ESM.docx]

Supplementary information

**Occurrence of the potent mutagens 2- nitrobenzanthrone and 3-nitrobenzanthrone in fine airborne particles**

**Aldenor G. Santos**^1,2+^**, Gisele O. da Rocha**^1,2,3+^**, Jailson B. de Andrade.**^1,2,3,4+*^

_1_Instituto de Química, Universidade Federal da Bahia, Campus de Ondina, 40170-115, Salvador-BA, Brazil

^2^Instituto Nacional de Ciência e Tecnologia em Energia e Ambiente - INCT, Universidade Federal da Bahia, 40170-115 Salvador, BA, Brazil

^3^Centro Interdisciplinar em Energia e Ambiente - CIEnAm, Universidade Federal da Bahia, 40170-115 Salvador, BA, Brazil.

^4^SENAI-CIMATEC University Center, 41650-110, Salvador, Bahia, Brazil

* jailsondeandrade@gmail.com

+these authors contributed equally to this work

1. Description of the analytical method

In the analytical point of view, a detailed method development and validation was performed, which includes a miniaturized extractor where it is possible to use sample filter pieces as small as 4.15 cm² with 500 µL dichloromethane / acetonitrile mix for quantitative extraction, as described in *Santos et al., (2016).* In the extraction procedure it was utilized a micro-extraction device (Whatmann MiniTM UniPrep Filters, Whatmann, USA). The syringeless micro-extractor consists of two pieces, a chamber with 0.5 mL capacity and a plunger, which are assembled one into the other during the extraction step. The plunger contains a PTFE filtration membrane (0.22 µm pore size) at one end and a lid with a pre-attached septum on the other end. For extraction, the plunger was first de-attached from the chamber then, a 4.15 cm^2^ filter pieces was cut into parts less than 1 cm long and transferred into the micro-extractor chamber. Following, it was added 500 µL ACN:DCM mixture (18% of acetonitrile in dichloromethane) onto the sample pieces and this compartment was closed with the micro-extractor plunger, which was used as a lid. Then, the whole micro-extractor system was sonicated during 23 min. After that, the plunger was manually pressed down into the micro-extractor chamber allowing the sample extract to go through the plunger membrane, filtrating the sample. It was not necessary any further preconcentration, sample fractionation or cleanup steps prior analysis. At this point, the micro-extractor was placed in the GC autosampler for direct injection in the GC–MS system. It is analyzed using a gas chromatographic method that in a 38 min total runtime we are able to determine a list of 52 polyaromatics in atmospheric samples. GC-MS was equipped with an AOC-20i autosampler (Shimadzu, Japan) and a Rtx® 5MS gas capillary column (crossbond® 5% diphenyl, 95% dimethylpolysiloxane, 30m×0.250mm×0.25µm) (from Restek Bellefonte, USA). During analysis, ultra-pure helium (99.999%) (WhiteMartins, Brazil) was used as carrier gas. Oven temperature program initiated at 70 ◦ C, it was held for 2 min, then 70^o^C to 200 ◦C at 30 ◦C min^-1^ and held for 5 min, then 200^o^C to 330 ◦ C, at 5 ◦ C min−1 and maintained for 0.67 min. Total running time was 38 min. The carrier gas was helium supplied at a flow rate of 1.0 mL min^-1^. The injector temperature was 310◦C at split/splitless mode, with a sampling time of 0.8 min. The mass spectrometer was operated in electron impact mode (EI), at 70eV. The ion source temperature was held at 250◦C, and the GC–MS transfer line temperature was set at 280◦C. The analysis was performed in simultaneous high-speed full scan and SIM modes. This single quadrupole mass spectrometer detector (MSD) performed simultaneous high-speed Scan/SIM data acquisition, providing ultra-trace level quantification and mass spectrum information. It is a comprehensive, efficient, fast, and clean method. In our analysis protocol it is included several QA/QC controls, the use of internal and surrogate standards and the monitoring of 2 to 3 different *m/*z signals for each analyte in order to approach unequivocal identification. Every detail of our procedure is validated through the use air dust reference material NIST SRM 1649b, traceable NIST standards, and the application to real samples. On the other hand, in *Santos et al., (2016)* there was not included 2-NBA since the pure analytical standard become available only afterwards. So, in the present manuscript we describe the insertion of this species and the differentiation from its isomer 3-NBA and the respective analysis method validation.


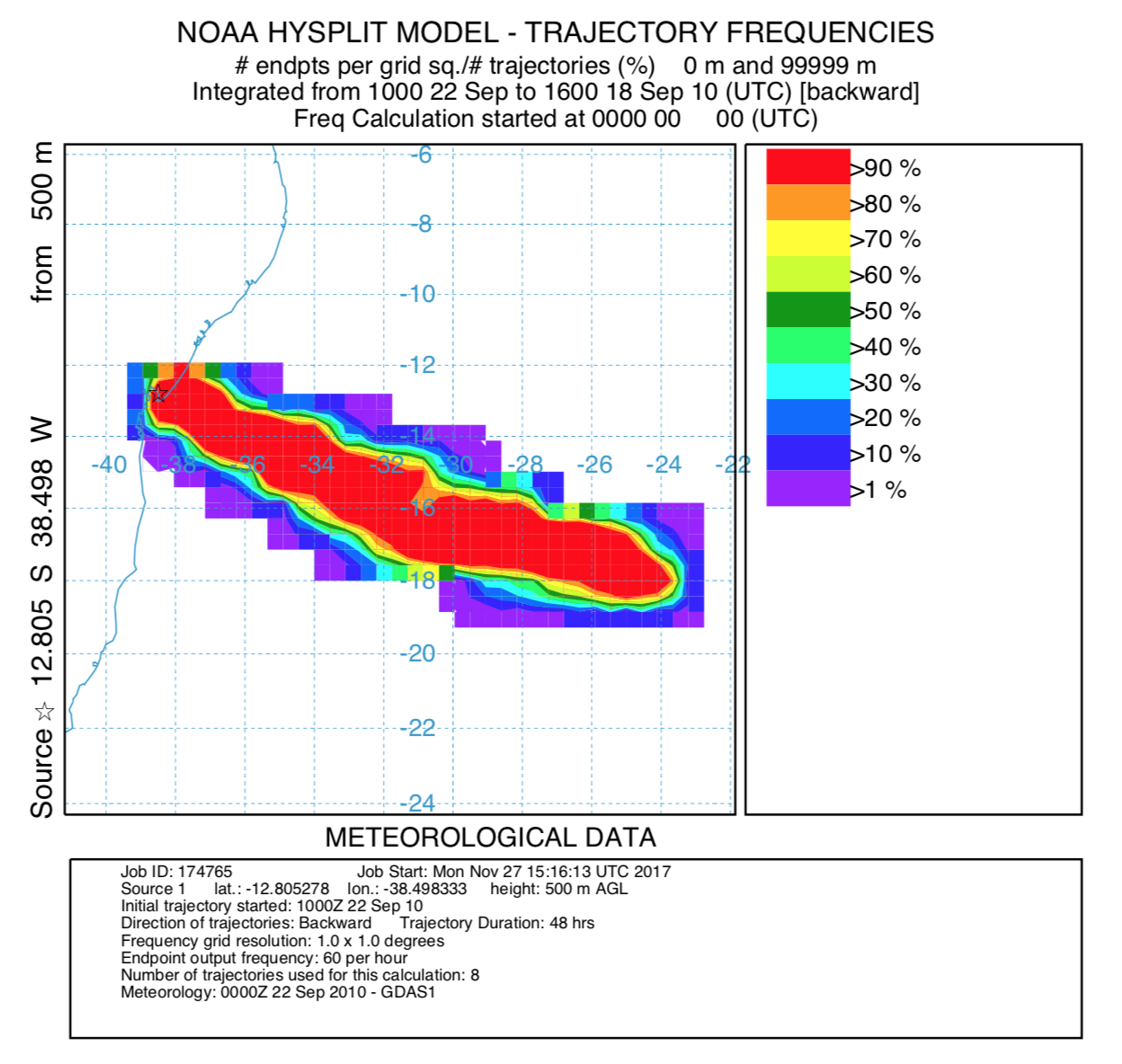


**Figure S1.** Backward air mass trajectory frequency (as number of endpoints per grid per number of trajectories) starting 96 h before arrive time (00:00 UTC) and altitudes ranging 0-99999 m a.g.l., for the coastal site at 22^nd^ Sept 2010.

**Figure S2.** GC–MS-SIM chromatogram of a real sample from the coastal site.

**Figure S3.** Electron impact mass spectra of selected nitro-PAHs and PAHs.

**Table S1**. Comparisons of selected PAHs and nitro-PAHs among previously published studies and the present investigation.

| **references** | **sample type (unit)** | **sampling site** | **FLT** | **PYR** | **BaP** | **BaA** | **1-NPYR** | **2-NPYR** | **2-NFLT** | **3-NFLT** | **2-NBA** | **3-NBA** |
| --- | --- | --- | --- | --- | --- | --- | --- | --- | --- | --- | --- | --- |
| *this study* | *PM2.5 (ng m-³)* | *bus station* | *2.03* | *3.84* | *0.52* | *0.85* | *1.02* | *0.39* | *0.38* | *1.31* | *-^a^* | *0.43* |
| *this study* | *PM2.5 (ng m-³)* | *coastal area* | *0.12* | *0.10* | *0.32* | *0.08* | *0.41* | *0.16* | *0.10* | *0.33* | *0.20* | *0.06* |
| Inazu et al., (2008) | PM10 (PAHs pmol m-³) | urban, spring | 0.3 | 0.4 | 0.2 | - | 16 | - | 164 | - | 233 | 0.7 |
|  | (NPAHs fmol m-³) | urban, summer | 0.3 | 0.6 | 0.6 | - | 21 | - | 148 | - | 215 | 1.4 |
|  |  | urban, autumn | 0.5 | 0.9 | 0.4 | - | 50 | - | 355 | - | 387 | 1.6 |
|  |  | urban, winter | 1.8 | 3.2 | 1.3 | - | 66 | - | 345 | - | 311 | 2.1 |
| Feilberg et al., (2002) | TSP (pg m-³) | semi-rural | - | - | - | - | - | - | - | - | - | 17.1 |
| Tang et al., (2004) | TSP (pg m-³) | roadside | - | - | - | - | 0.3 | - | - | - | 1.83 | 24.7 |
| Murahashi et al., (2003) | rainwater (ng L-¹) | urban | - | - | - | - | 0.055 | - | - | - | - | 0.81 |
| Murahashi et al., (2003) | TSP (pg mg-¹) | diesel exhaust | - | - | - | - | - | - | - | - | - | 27-56 |
| Hasei et al., (2012) | incinerator dust (pg g-¹) | - | - | - | - | - | - | - | - | - | - | 26 |
|  | TSP (pg m-³) | urban | - | - | - | - | - | - | - | - | - | 7.7 |
| Kameda et al., (2004) | TSP (PAHs pmol m-³) (NPAHs fmol m-³) | roadside, site I^b^ | 21.5 | 37.5 | 6.0 | - | 171 | 35 | 167 | 5.0 | - | - |
|  |  | sub-urban, site S^b^ | 6.4 | 3.9 | 1.9 | - | 29 | 14.5 | 97 | 1.4 | - | - |
| Enya et al., (1997) | TSP (µg g-¹) | diesel exhaust | - | - | - | - | - | - | - | - | - | <0.001-6.61 |
|  | TSP (pg m-³) | urban | - | - | - | - | - | - | - | - | - | 7.7 |
| Albinet et al., (2007) | PM10 + vapor | urban | 3.49 | 5.83 | 0.10 | 0.077 | 0.061 | 0.034 | - | 0.090^c^ | - | - |
|  | (ng m-³) | sub-urban | 0.89 | 0.60 | 0.058 | 0.044 | 0.079 | 0.010 | - | 0.024 | - | - |
|  |  | rural | 1.00 | 12.78 | 0.018 | 0.009 | 0.006 | 0.002 | - | 0.003 | - | - |
| Dvorská et al., (2012) | TSP + vapor | background, winter | 1.86-7.99 | 1.29-5.32 | 0.29-1.15 | 0.33-1.84 | - | - | - | - | - | - |
|  | (ng m-³) | background, summer | 0.25-1.02 | 0.14-0.95 | 0.02-0.11 | 0.02-0.09 | - | - | - | - | - | - |
| Huang et al., (2014) | TSP (ng m-³) | sub-urban | 1.61 | 1.62 | 1.63 | 0.93 | 0.017 | 0.11 | 1.55 | 0.003 | - | - |
| Harrison et al., (2016) | TSP + vapor | coast, site C | 0.94 | 0.89 | 0.07 | 0.26 | 0.34 | 0.16 | 0.78 | - | - | - |
|  | (ng m-³) | coast, site D | 1.20 | 1.44 | 0.09 | 0.39 | 0.55 | 0.13 | 0.50 | - | - | - |
|  |  | coast, site E | 1.18 | 1.11 | 0.16 | 0.37 | 0.61 | 0.23 | 0.59 | - | - | - |
| Keyte et al., (2016) | PM10 + vapor | tunnel #1 | 13.6 | 11.1 | 0.9 | 1.0 | 343 | 15 | 9 | 18^c^ | - | - |
|  | (ng m-³) | tunnel #2 | 6.8 | 7.5 | 0.8 | 0.7 | 287 | nd | 19 | nm | - | - |
|  |  | tunnel #3 | 3.0 | 3.7 | 0.1 | 0.1 | 5 | 6 | 11 | 1 | - | - |
| Lammel et al., (2017) | TSP + vapor | marine | 226 | 158 | - | 2.8 | 0.75 | 2.5 | 8.6^c^ | - | - | - |
|  | (pg m-³) | continental | 342 | 226 | - | 16 | 1.1 | 1.3 | 15^c^ | - | - | - |
| Hayakawa et al., (2018) | TSP  (pmol m-³ for PAH and fmol m-³ for NPAH) | urban, 5 cities | ND^d^-8.7 | 0.13-8.7 | 0.11-3.6 | 0.067-5.0 | 3.6-570 | - | - | - | - | - |
| Jiang et al., (2018) | (PM2.5 + PM10)  (pg m-³) | haze | - | - | - | - | 38.4 | 130.9 | 759^c^ | - | - | - |
|  |  | dust storm 1 | - | - | - | - | 18.7 | 25.2 | 2412 | - | - | - |
|  |  | dust storm 2 | - | - | - | - | 26.3 | 67.6 | 584 | - | - | - |
|  |  | dust storm 3 | - | - | - | - | 10.9 | 10.9 | 326 | - | - | - |
|  |  | clear | - | - | - | - | 3.64 | 7.25 | 61.4 | - | - | - |
| Galvão et al., (2018) | PM10  (pg m-³) | biomass burning | - | - | - | - | 125 | - | - | - | - | - |
| Zhang et al., (2018) | PM2.5 (pg m-³) | urban, summer | - | - | - | - | 11.8 | 57.5 | 328 | - | - | - |
|  |  | urban, winter | - | - | - | - | 145 | 631 | 2510 | - | - | - |
| Zhao et al., (2018) | PM2.5 (pg m-³) | urban, with traffic | - | - | - | - | 17.7-59.6 | - | 42.9-67.1 | - | - | 191.2-253.5 |
|  |  | urban, no traffic | - | - | - | - | 14.2-72.7 | - | 21.5-75.1 | - | - | 70.4-267.3 |

^a^ not analyzed, ^b^ mean between summer and winter, ^c^ 2- + 3-nitrofluoranthene, ^d^ not detected.

**Table S2.** Physical-chemical properties of selected compounds.^1^

| compound | abbreviation | molecular weight (g mol-¹) | boiling point (^o^C) | vapor pressure (mmHg) | log K_ow_ | log K_oa_ |
| --- | --- | --- | --- | --- | --- | --- |
| 1-nitropyrene | 1-NPYR | 249.248 | 428 | 6.90 x 10^-8^ | 2.55 | 9.32 |
| 2-nitropyrene | 2-NPYR | 249.248 | 428 | 4.00 x 10^-8^ | 2.55 | 9.32 |
| 2-nitrofluoranthene | 2-NFLT | 247.248 | 424 | 4.02 x 10^-8^ | 2.55 | 9.32 |
| 3-nitrofluoranthene | 3-NFLT | 247.248 | 427 | 6.91 x 10^-8^ | 2.55 | 9.32 |
| 2-nitrobenzanthrone | 2-NBA | 275.258 | -^2^ | -^2^ | 3.90 | -^2^ |
| 3-nitrobenzanthrone | 3-NBA | 275.258 | 506.2 | 5.05 x 10^-9^ | 3.99 | 12.51 |
| benzanthrone | BA | 230.266 | 417 | 3.28 x 10^-8^ | 4.81 | 9.69 |
| fluoranthene | FLT | 202.251 | 375 | 5.50 x 10^-6^ | 5.16 | 8.88 |
| pyrene | PYR | 202.251 | 399 | 4.50 x 10^-6^ | 4.88 | 8.80 |
| benzo(a)pyrene | BaP | 252.309 | 495 | 2.44 x 10^-6^ | 5.99 | 10.3 |
| benzo(a) anthracene | BaA | 228.288 | 437 | 4.50 x 10^-6^ | 5.76 | 9.37 |

^1.^ Sources: [www.comptox.epa.gov/dashboard](http://www.comptox.epa.gov/dashboard); [www.chemspider.com](http://www.chemspider.com).

^2^ data not available for 2-NBA, we assumed boiling point, vapor pressure and logK_oa_ for 2-NBA are similar to the 3-NBA values in this study.

**Table S3.** Principal component analysis (PCA) scores for (a) bus station and (b) coastal site.

**(a)**

| variables | PC1 (34.7 %) | PC2 (32.2 %) |
| --- | --- | --- |
| mass | **0.859** | 0.166 |
| mass conc | **0.883** | -0.015 |
| 3NBA | **-0.539** | 0.052 |
| 3NFLT | **-0.851** | -0.396 |
| 1NPYR | **0.425** | -0.410 |
| FLT | -0.196 | **0.928** |
| PYR | -0.101 | **0.928** |
| BaA | -0.124 | **0.883** |
| BaP | **-0.586** | -0.208 |

**(b)**

| variables | PC1 (54.1 %) | PC2 (14.9 %) | PC3 (13.6 %) |
| --- | --- | --- | --- |
| mass | **-0.936** | -0.001 | 0.282 |
| mass conc | **-0.934** | 0.004 | 0.303 |
| 2NBA | **0.791** | -0.169 | -0.392 |
| 3NBA | 0.610 | **0.724** | -0.088 |
| 2NFLT | **0.865** | -0.126 | -0.041 |
| 3NFLT | **0.946** | -0.236 | -0.104 |
| 1NPYR | **0.636** | -0.611 | 0.318 |
| 2NPYR | **0.841** | -0.207 | 0.194 |
| FLT | 0.694 | **0.507** | 0.427 |
| PYR | 0.699 | **0.436** | 0.521 |
| BaA | 0.080 | -0.476 | **0.738** |
| BaP | -0.101 | 0.279 | **0.391** |

**Table S4.** Data inputs for calculation of (a) daily inhalation exposure (E_I_) and (b) incremental lifetime cancer risk (ILCR).

**(a)**

| **Target compounds** | **TEF*** | **Ref** | **MEF^+^** | **Ref** |
| --- | --- | --- | --- | --- |
| 2-nitrobenzanthrone¹ | 0.031 | OEHHA, (2011) | 1.2 | Durant et al., (1996) |
| 3-nitrobenzanthrone² | 1 | OEHHA, (2011) | 1.8 | Durant et al., (1996) |
| 2-nitrofluoranthene | 0.05 | Durant et al., (1996) | 0.05 | Durant et al., (1996) |
| 3-nitrofluoranthene³ | 0.05 | Durant et al., (1996) | 0.0026 | Durant et al., (1996) |
| 1-nitropyrene | 0.1 | OEHHA, (2011) | 0.025 | Durant et al., (1996) |
| 2-nitropyrene^4^ | 0.1 | OEHHA, (2011) | -^#^ | Durant et al., (1996) |
| Fluoranthene | 0.001 | Nisbet and LaGoy, (1992) | - | Durant et al., (1996) |
| Pyrene | 0.001 | Nisbet and LaGoy, (1992) | - | Durant et al., (1996) |
| Benz[a]anthracene | 0.1 | Nisbet and LaGoy, (1992) | 0.082 | Durant et al., (1996) |
| benzo[a]pyrene | 1 | Nisbet and LaGoy, (1992) | 0.055 | Durant et al., (1996) |

* toxic equivalent factors relative to benzo[a]pyrene.

^+^ mutagenic equivalent factors relative to benzo[a]pyrene.

¹ it was used the TEF and MEF from 1,3 dinitropyrene due to comparable carcinogenicity and mutagenicity.

² it was used the TEF and MEF from 1,8 dinitropyrene due to comparable carcinogenicity and mutagenicity.

³ it was used the TEF from 2NFLT since they are isomers.

^4^ it was used the TEF from 1-NPYR and 4-NPYR since they are isomers.

# not considered mutagenic due to limited experiments and/or not enough data for elucidation.

**(b)**

| **Target population*** | **IR**  **(m³ day-¹)** | **BW**  **(kg)** | **EF**  **(day year-¹)** | **E_D_**^#^  **(year)** |
| --- | --- | --- | --- | --- |
| adults (> 21 years) | 16.4 | 80 | 365 | 50 |
| adolescents (11-16 years) | 21.9 | 56.8 | 365 | 6 |
| children (1-11 years) | 13.3 | 26.5 | 365 | 11 |
| infants (< 1 year) | 6.8 | 6.8 | 365 | 1 |
| *Source: US Environmental Protection Agency. Exposure factors handbook. www.epa.gov/data/ncea/efh/pdfs/efh-complete.pdf, 2011. (accessed 15^th^ Dec 2017). | | | | |

^#^ it is the lifespan between the lower limit and the higher limit of the age range for each group, up to 70 years (according to ILCR calculations). For instance, for adults E_D_ = 70 – 21 years = 50 years (including the age 21 and 70) and for adolescents E_D_ = 16 - 11 years = 6 years (including the age 11 and 16), and so on

**References**

Albinet, A.; Leoz-Garziandia, E.; Budzinski, H.; Villenave, E. Polycyclic aromatic hydrocarbons (PAHs), nitrated PAHs and oxygenated PAHs in ambient air of the Marseilles area (South of France): concentrations and sources. *Sci. Total Environ*. **2007**, *384*, 280-292.

ChemSpider Search and Share chemistry, [www.chemispider.com](http://www.chemispider.com), accessed on 31^st^ August 2018.

Durant, J. L.; Busby Jr., W. F.; Lafleur, A. L.; Penman, B. W.; Crespi, C. L. Human cell mutagenicity of oxygenated, nitrated and unsubstituted polycyclic aromatic hydrocarbons associated with urban aerosols. *Mutat. Res.* **1996**, *371*, 123-157.

Dvorská, A.; Komprdová, K.; Lammel, G.; Klánová, Plachá, H. Polycyclic aromatic hydrocarbons in background air in central Europe – Seasonal levels and limitations for source apportionment. *Atmos. Environ*. **46**, 147-154 (2012).

Enya, T.; Suzuki, H.; Watanabe, T.; Hirayama, T.; Hisamatsu, Y. 3-Nitrobenzanthrone, a powerful bacterial mutagen and suspected human carcinogen found in diesel exhausts and airborne particulates. *Environ. Sci. Technol.* **1997**, *31***,** 2772–2776.

Feilberg, A.; Ohura, T.; Nielsen, T.; Poulsen, M. W. B.; Amagai, T. Occurrence and photostability of 3-nitrobenzanthrone associated with atmospheric particles. *Atmos. Environ*. **2002**, *36*, 3591–3600.

Galvão MFO, Alves NO, Ferreira PA, Caumo S, Vasconcellos PC, Artaxo P, Hacon SS, Roubicek DA, Medeiros SRB. Biomass burning particles in the Brazilian Amazon region: mutagenic effects of nitro and oxy-PAHs and assessment of health risks. Environmental Pollution, 233, 960-970, 2018.

Harrison, R. M.; Alam, M. S.; Dang, J.; Ismail, I. M.; Basahi, J.; Alghamdi, M. A.; Hassan, I. A.; Khoder, M. Relationship of polycyclic aromatic hydrocarbons with oxy(quinone) and nitro derivatives during air mass transport. *Sci. Total Environ.* (2016) doi: 10.1016/j.scitotenv.2016.08.030.

Hasei, T.; Nakanishi, H.; Toda, Y.; Watanabe, T. Development of a two-dimensional high-performance liquid chromatography system coupled with on-line reduction as a new efficient analytical method of 3-nitrobenzanthrone, a potential human carcinogen. *J. Chromatog. A* **2012**, *1253*, 52-57.

Hayakawa K, Tang N, Nagato EG, Toriba A, Sakai S, Kano F, Goto S, Endo O, Arashidani K, Kakimoto H. Long term trends in atmospheric concentrations of polycylic aromatic hydrocarbons and nitropolycyclic atomatic hydrocarbons: a study of Japanese cities from 1997 to 2004. Environmental Pollution, 233, 474-482, 2018.

Huang, B.; Liu, M.; Bi, X.; Chaemfa, C.; Ren, Z.; Wang, X.; Sheng, G.; Fu J. Phase distribution, sources and risk assessment of PAHs, NPAHs and OPAHs in a rural site of Pearl River Delta region, China. *Atmos. Poll. Res****.* 2014**, *5*, 210-218.

Inazu, K.; Nam, V. D.; Asato, T.; Okoshi, H.; Hisamatsu, Y.; Kobayashi, T.; Baba, T. Atmospheric occurrence of 2-nitrobenzanthrone associated with airborne particles in central Tokyo. *Polycycl. Aromat. Compd.* **2008**, *28*, 562-577.

Jiang, P.; Yang, L.; Chen, X.; Gao, Y.; Li, Y.; Zhang, J.; Zhao, T.; Yu, H.; Wang, W. Impact of dust storms on NPAHs and OPAHs in PM2.5 in Jinan, China, in Spring 2016: Concentrations, health risks, and sources, *Aerosol Air Qual. Res*. **18,** 471-484, 2018.

Kameda, T.; Takenaka, N.; Bandow, H.; Inazu, K.; Hisamatsu, Y. Determination of atmospheric nitro-polycyclic aromatic hydrocarbons and their precursors at a heavy traffic roadside and at a residential area in Ozaka, Japan. *Polycycl. Aromat. Compd.* **2004**, *24*, 657-666.

Keyte, I. A., Albinet, A. & Harrison, R. M. On-road traffic emissions of polycyclic aromatic hydrocarbons and their oxy- and nitro- derivative compounds measured in road tunnel environments. *Sci. Total Environ.* **566-567**, 1131-1142 (2016).

Lammel, G.; Mulder, M. D.; Shahpuory, P.; Kukuchka, P.; Lisková, H.; Pribylová, P.; Prokes, R.; Wotawa G. Nitro-polycyclic aromatic hydrocarbons – gas-particle partitioning, mass size distribution, and formation along transport in marine and continental background air. *Atmos. Chem. Phys.* **17**, 6257-6270 (2017).

Murahashi, T. Determination of mutagenic 3-nitrobenzanthrone in diesel exhaust particulate matter by three-dimensional high-performance liquid chromatography. *Analyst* **2003**, *128*, 42-45.

Murahashi, T.; Iwanaga, E.; Watanabe, T.; Hirayama, T. Determination of the mutagen 3-nitrobenzanthrone in rainwater collected in Kyoto, Japan. *J. Health Sci.* **2003**, *49*, 386-390.

Nisbet, I. C. T.; LaGoy, P. K. Toxic equivalency factors (TEFs) for polycyclic aromatic hydrocarbons (PAHs). *Regul. Toxicol. Pharmacol.* **1992**, *16*, 290-300.

NOAA HYSPLIT database, 2017 (<http://ready.arl.noaa.gov/HYSPLIT.php)> (Accessed December 1^st^, 2017).

### OEHHA, Office of Environmental Health Hazard Assessment. Chemical-specific summaries of the information used to derive unit risk and cancer potency values. Appendix B. 2011. <http://www.oehha.ca.gov/hot_spots/tsd052909.html> (accessed December 15th, 2017).

Tang, N.; Taga, R.; Hattori, T.; Tamura, K.; Toroba, A.; Kizu, R.; Hayakawa, K. Determination of atmospheric nitrobenzanthrones by high-performance liquid chromatography with chemiluminescence detection. *Anal. Sci*. **2004**, *20*, 119–123.

US EPA CompTox Dashboard, <http://comptox.epa.gov/dashboard>, accessed on 31^st^ August 2018.

USEPA, US Environmental Protection Agency. Exposure factors handbook. 2011. [www.epa.gov/data/ncea/efh/pds/efh-complete.pdf](http://www.epa.gov/data/ncea/efh/pds/efh-complete.pdf) (accessed December 15th, 2017).

Zhang, J. *et al* Atmospheric PAHs, NPAHs, and OPAHs at an urban, mountainous, and marine sites in Northern China: Molecular composition, sources, and ageing. *Atmos. Environ*. **173**, 256–264 (2018). doi: 10.1016/j.atmosenv.2017.11.002

Zhao J, Zhang J, Sun L, Liu Y, Lin Y, Li Y, Wang T, Mao H. Characterization of PM2.5-bound nitrated and oxygenated polycyclic aromatic hydrocarbons in ambient air of Langfang during periods with and without traffic restriction. Atmospheric Research, 213, 302-308, 2018.
